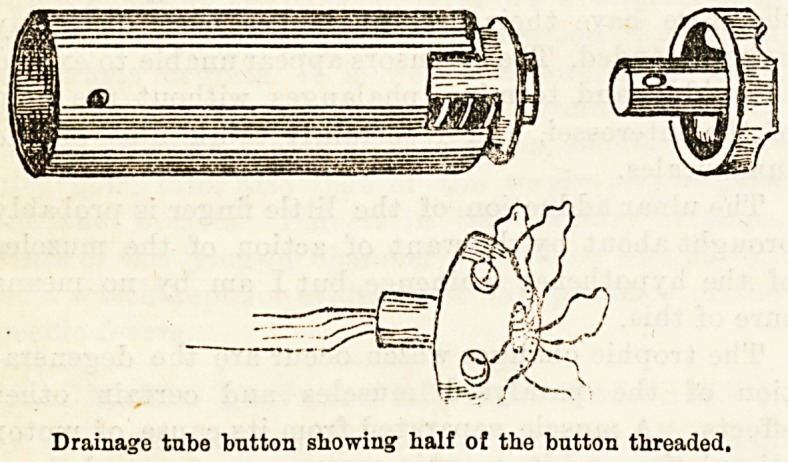# Surgery of the Liver and Gall Bladder

**Published:** 1894-12-01

**Authors:** 


### PROGRESS IN SURGERY.

### SURGERY OF THE LIVER AND GALL BLADDER.

(*Continued from page* 138.)

**Operations on the Gall-Bladder.**—Dr. J. B. Murphy records[5] twenty successive Cholecystenterostomies by means of his anastomosis button. He divides operations on the gall-bladder into ten classes:[6] (1) Puncture of the gall-bladder. (2) Incision into the gall-bladder without further operative procedure. This operation is limited to those cases in which gangrene of the wall of the gall-bladder or adhesions around it prevent suturing to the abdominal wall, or approximating to the intestine, and where drainage is necessary. The writer recommends the "button-tube," and the operation is performed as follows: An incision is made in the abdominal wall, beginning at the ninth costal cartilage, parallel to the external border of the rectus muscle, for a distance of two and a-half inches. A sufficient surface of the wall of the gall-bladder is exposed, the contents aspirated, the purse-string suture inserted, the gall-bladder incised, male half of button inserted, purse-string tied and cut short, and the tubular portion of the button is then pressed into position; the tube is then drawn out as far as the gall bladder will permit, and held there with a pin passed through the openings in the side. During the time the "pressure atrophy" in the portion of the gall bladder clasped between the two halves of the button is taking place, a cicatricial wall is being formed about the tube, which acts as the walls of a sinus after its production, and insures continued protection to the peritoneal cavity. (3) Suture of the gall-bladder to the abdominal wall with secondary incision, cholecystostomy in two sittings. (4) Suture of the gall bladder to the abdominal wall with immediate incision. Murphy thinks the anastomosis button is much safer and quicker than the suture.

Drainage tube button showing half of the button threaded.

Half of the button is threaded by passing two pieces of surgeon's silk about eighteen inches long through the four drainage openings in the bowl of the button, each thread being passed through two of the openings nearest one another; the four ends are passed through the cylinder of the button, entering the cylinder at its junction with the bowl. By this means traction may be made and the two halves of the button approximated. The threaded half of the button is inserted in the gall bladder in the same manner as in cholecystenterostomy; an artery forceps is then pushed through the parietal layer of the peritoneum, one half-inch to the side of the incision, grasping the stem of the button inserted in the gall bladder, and drawing the stem through the opening made by the artery forceps, the traction cord is passed through the other half of the button, the button drawn together and the threads removed. He sews up the opening in the peritoneum with catgut. (5) Incision of the gall-bladder, removing its contents, suturing it to the abdominal wall, with immediate extra-peritoneal suture of the incision made in the gall-bladder. (6) Cholecystendysis. Incision of gall bladder, with immediate suture and reposition into abdominal cavity. (7) Cholecystenterostomy, or gall-bladder and intestinal anastomosis. For this the author uses his small anastomosis button. The atrophy produced by the pressure of the spring cup leaves an opening large enough to allow the cup to drop into the bowel, and it is passed through the intestine. (8) Cholecystectomy. Total extirpation of the gall-bladder. (9) Choledocholithotripsy, or crushing of gall stones in the choledochus. (10) Choledocholithectomy, with subsequent suture of duct. Instead of the operation of cholecystostomy, the author recommends[7] the performance of cholecystenterostomy whenever it can be done. Cholecystendysis is not to be recommended, for of thirty-five cases collected, eight (23 per cent.) terminated fatally. The mortality is greatest in the operation of cholecystotomy at one sitting by means of suture, in which 30 per cent. terminated fatally. Cholecystostomy at one sitting (which is the operation most frequently performed) has a mortality of 19 per cent., while when performed at two sittings it is 10 per cent. In cholecystectomy the mortality is 17 per cent., and in cholecystenterostomy with suture 35 per cent., whilst when the latter was performed by the author with his anastomosis button, the recoveries were 100 per cent. Dr. C. C. Barrows also reports[8] a successful case of cholecystenterostomy with the Murphy button. The button was passed on the ninth day.

**Rupture of the Common Bile Duct.**—A boy who had been run over came under Mr. Battle's care with the usual course of symptoms in these cases[9]; severe injury followed by shock, and few definite abdominal signs until after some days, and then the appearance of fluid in the abdomen. He operated on the eighth day, but the condition of the patient was too bad to allow of a prolonged search for the point of injury. He died on the ninth day, and, *post mortem*, a complete transverse rupture of the common bile duct without laceration of the liver was found.

**Biliary Lithiasis Treated by Injections of Ether.**—Dr. Fontan has had under observation a woman of thirty-three, who for several months was affected with persistent jaundice, consequent upon hepatic crises[10]. After two months internal treatment with no effect, it was decided to perform cholecystotomy. More than thirty calculi were removed from the gall-bladder, and cystic duct. When the gall-bladder was solidly united to the abdominal parietes, the obstructions of the bile ducts were cleared away by catheterism combined with injections of ether. By this means the bile was made to flow first through the fistula and then through the regular channels into the intestine. The circulation of the bile now persists, the fæces have resumed their normal colour, and the patient has gained strength. Dr. Routier thinks[11] that cholecystostomy as a surgical operation for the treatment of biliary lithiasis is preferable to cholecystenterostomy on account of the risk of ascending infection which may result from the latter operation. Professor Terrier[12] regards this, however, as merely a theoretical objection.

[5] Chicago Medical Rec., Mar. and April. 1894. [6] Internat. Med. Mag., June, 1894, p. 367. [7] Med. Rec., Jan. 13th and 20th, 1894; and Brit. Med. Journ., Mar. 31st, 1894. [8] N. Y. Med. Journ., April 21st, 1894, p. 503. [9] Brit. Med. Journ., April 7th, 1894. [10] Med. Week, July 13th, 1894, p. 330. [11] Med. Week, July 27th, 1894, p. 354. [12] Ibidem.

## Figures and Tables

**Figure f1:**